# Assessing Risk Factors for Cognitive Decline Using Electronic Health Record Data: A Scoping Review

**DOI:** 10.21203/rs.3.rs-4671544/v1

**Published:** 2024-08-09

**Authors:** Liqin Wang, Richard Yang, Ziqin Sha, Anna Maria Kuraszkiewicz, Conrad Leonik, Li Zhou, Gad A. Marshall

**Affiliations:** Brigham and Women’s Hospital; Brigham and Women’s Hospital; Lexington High School; University of Massachusetts Amherst; Louisiana State University School of Medicine; Brigham and Women’s Hospital; Brigham and Women’s Hospital

**Keywords:** Alzheimer Disease, Dementia, Cognitive Dysfunction, Risk Factors, Electronic Health Records

## Abstract

**Background:**

The data and information contained within electronic health records (EHR) provide a rich, diverse, longitudinal view of real-world patient histories, offering valuable opportunities to study antecedent risk factors for cognitive decline. However, the extent to which such records’ data have been utilized to elucidate the risk factors of cognitive decline remains unclear.

**Methods:**

A scoping review was conducted following the PRISMA guideline, examining articles published between January 2010 and April 2023, from PubMed, Web of Science, and CINAHL. Inclusion criteria focused on studies using EHR to investigate risk factors for cognitive decline. Each article was screened by at least two reviewers. Data elements were manually extracted based on a predefined schema. The studied risk factors were classified into categories, and a research gap was identified.

**Results:**

From 1,593 articles identified, 80 were selected. The majority (87.5%) were retrospective cohort studies, with 66.3% using datasets of over 10,000 patients, predominantly from the US or UK. Analysis showed that 48.8% of studies addressed medical conditions, 31.3% focused on medical interventions, and 17.5% on lifestyle, socioeconomic status, and environmental factors. Most studies on medical conditions were linked to an increased risk of cognitive decline, whereas medical interventions addressing these conditions often reduced the risk.

**Conclusions:**

EHR data significantly enhanced our understanding of medical conditions, interventions, lifestyle, socioeconomic status, and environmental factors related to the risk of cognitive decline.

## BACKGROUND

Alzheimer’s disease (AD) presents a substantial global public health challenge, given its hallmark features of chronic cognitive and functional decline in older adults. The condition is commonly categorized into three stages based on cognitive impairment severity: preclinical, where individuals exhibit normal cognitive function with or without subtle concerns but have biological evidence of underlying AD; prodromal, marked by mild cognitive impairment (MCI); and the dementia stage, characterized by significant functional impairment affecting daily life.^1, 2^ As of 2023, a staggering 6.7 million Americans are living with AD in its dementia stage, with projections estimating this number to soar to 88 million by 2050.^3^ This not only poses a substantial financial burden but also profoundly impacts affected individuals, their families, and the healthcare system. Consequently, there is an urgent need to comprehensively grasp the risk factors associated with dementia and identify potential prevention and treatment strategies to mitigate this growing concern.

Existing studies have frequently relied on prospective datasets, which tend to suffer from limitations such as small sample sizes and underrepresentation of understudied populations, resulting in notable gaps in ADRD research.^4, 5^ There is a growing consensus in the scientific community on the necessity of exploring more extensive and diverse populations.^1^

Electronic Health Record (EHR) data have proven pivotal in understanding the progression and outcomes of neurodegenerative diseases, particularly due to their chronic and gradually advancing nature. The widespread adoption of EHRs over recent decades has yielded a vast amount of longitudinal patient data. By sifting through these real-world datasets, we can gain deeper insights into the onset and evolution of AD and related dementias (ADRD), especially among populations that have been consistently engaged with the healthcare system. EHRs can be valuable in identifying potential risk factors for ADRD that might be missed in smaller convenience sample datasets. Moreover, they can highlight interventions that target certain medical problems that potentially affect the risk of dementia, particularly during early stages such as preclinical AD and MCI.

However, the extent to which EHR data have been utilized for such research remains unclear. While prior literature reviews have primarily focused on specific ADRD risk areas,^6–8^ none, to our knowledge, has specifically addressed the utilization of EHR data for analyzing ADRD risks. Our study aims to fill this gap by concentrating on the identification of risk factors for cognitive decline, with a specific emphasis on MCI and dementia. We have intentionally excluded the preclinical stage of cognitive decline from our analysis due to diagnostic challenges in the clinical setting where biomarkers of AD are not commonly obtained prior to the stage of MCI. Through this scoping review, we aim to thoroughly aggregate existing literature on EHR data usage for studying these stages of cognitive decline and highlight potential areas for future research.

## METHODS

### Search Strategy

This scoping review followed the Preferred Reporting Items for Systematic Reviews and Meta-Analyses (PRISMA) guidelines.^9^ We conducted a Boolean search in PubMed, Web of Science, and CINAHL, identifying English-language studies published between January 1, 2010, and April 30, 2023. Our search included keywords related to cognitive impairment stages, such as dementia, MCI, and normal cognition, as well as EHR-related terms like “electronic health records”. The specific queries for individual databases can be found in Supplementary Table 1. This study does not involve direct experimentation on human or animal subjects. All procedures and analyses comply with the ethical standards of the institution.

### Study Selection

We included studies that utilized EHR datasets to investigate the association between potential risk factors and dementia outcomes. The EHR datasets referred to data extracted from EHR systems, not the active EHR systems themselves. We excluded review articles without original data, non-English articles, studies focused on patients with preexisting cognitive impairment at baseline, those with small sample size (n < 100) or short follow-up times (< 1 year), non-epidemiology studies (e.g., algorithm evaluation), and studies of low-quality with missing or unclear components (e.g., unclear diagnostic criteria for outcomes).

### Screening Process

After eliminating duplicates and using automation tools (e.g., classification by the search engine, keyword-based search of the tile and abstract) to exclude articles deemed ineligible, we obtained abstracts from the search results. Two reviewers independently assessed titles and abstracts based on the inclusion and exclusion criteria, resolving disagreements through discussion to reach a consensus. Subsequently, two reviewers independently performed full-text screening, with a senior reviewer addressing any disagreements.

### Data Extraction

We extracted articles assessing risk factors for the cognitive decline onset, including MCI, AD, and other dementias. We assessed methodological quality and developed a data extraction schema based on the Strengthening the Reporting of Observational Studies in Epidemiology (STROBE) checklist for observational studies.^10^ Extracted data included article information (authors and year), objectives, study design (e.g., cohort or case-control), study cohort, sample size, follow-up duration, data sources, explored risk factors, confounding variables, outcomes and measurement, statistical methods, and key findings. Each article underwent independent extraction by two reviewers, with discrepancies resolved through discussions, or consultation with a third reviewer.

### Article Classification

After extracting risk factors from articles, we categorized them into major groups, including medical conditions, medical interventions, lifestyle, socioeconomic, psychosocial, and environmental factors. These groups were further subdivided; for example, medical conditions included cardiovascular and metabolic conditions, as well as psychiatric conditions. Some articles covered multiple risk factors, leading to overlap across categories.

## RESULTS

**Figure 1:**
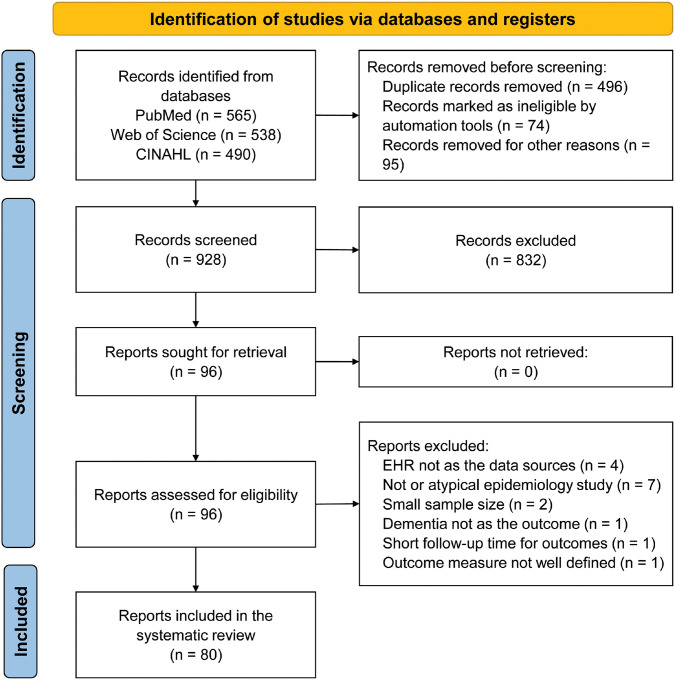
PRISMA flow diagram

Figure 1 shows the PRISMA flow diagram. The initial search yielded 1,593 articles, 565 from PubMed, 538 from Web of Science, and 490 from CINAHL. We removed 496 duplicate articles, where the same article appeared in more than one database. Automated tools marked 74 articles as ineligible, which included 3 case reports, 28 review articles, and 43 articles without abstracts. We also excluded 95 additional records, such as 42 datasets, 25 preprints, 13 authorless articles, 9 patents, 3 genetic studies, 2 books, and 1 thesis. Subsequently, during title and abstract screening, 832 articles were excluded for not meeting the criteria. The remaining 96 articles underwent full-text screening. After excluding additional 16 articles, e.g., those not primarily using EHR data, or having small sample size, 80 articles remained for final analysis. A detailed list of these articles and extracted data is available in **Supplementary Table 2** in the supplement.

### Research Trend Over Time

**Figure 2:**
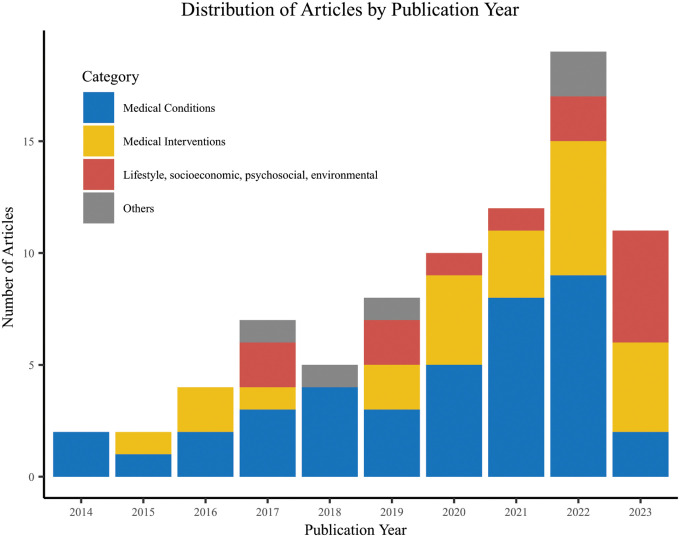
Distribution of articles by publication years, by type of risk factors. Four articles that were classified into more than one category have been counted under the medical condition category.^26, 32, 45, 61^

Figure 2 illustrates the distribution of analyzed articles by publication year. It shows a notable increase in publications related to our topic over the past decade, indicating a growing trend in using EHR data to examine ADRD risk factors. Although our search spanned from 2010 to 2023, all included articles were published after 2014. More than one-quarter of the articles (n = 22, 27.5%) were published in 2022. It is important to note that our search was conducted up to April 2023; therefore, the total for that year does not reflect the full annual count.

### Study Design

Out of the 80 articles reviewed, 77(96.3%) were longitudinal studies retrospectively conducted, comprising 70 cohort studies, six case-control studies, and one randomized control trial. Longitudinal studies had a median EHR duration of 16 years, calculated from the initial year to the final year of the EHR records utilized, regardless of individual patient follow-up time. Among these, 16 studies (20%) had EHR data spanning under 10 years, 39 studies (48.8%) ranged between 10 and 20 years, and 22 studies (27.5%) had data duration exceeding 20 years.

### Methods for Statistical Analyses

In the statistical analysis, 76.3% of the studies (n = 61) predominantly used survival analysis to model and identify various risk or protective factors. Among these, most (n = 54, 88.5%) opted for the Cox proportional-hazards regression model,^11^ while some (n = 13, 21%) used the Fine-Gray model,^12^ often in combination. The Fine-Gray model was chosen for its ability to handle competing risks like death. Other statistical analysis methods included logistic regression, Chi-squared test, and analysis of variance (ANOVA).

### EHR Datasets and Sources

The included articles utilized diverse datasets to examine ADRD risk factors. These datasets were derived either directly from EHR systems, such as Veterans Health Administration (VHA), or linked to EHR databases to incorporate specific variables or outcomes from external databases, such as the UK Biobank. Categorized by geographical location, almost half of the studies (46.3%, n = 37) used data from EHR systems within the United States (US), while 40% (n = 32) utilized datasets from the United Kingdom (UK). Additional countries represented in this review included Australia (n = 3),^13–15^ China (n = 3),^16–18^ Denmark (n = 3),^19–21^ the Netherlands (n = 3),^20–22^ Taiwan (n = 2),^23, 24^ Canada (n = 2),^25, 26^ and Sweden (n = 2).^27, 28^

In the US, the most frequently used EHR dataset was derived from the Kaiser Permanente’s EHR (11 studies), followed by the VHA (6 studies). The remaining 21 articles used databases from other US healthcare systems and commercial sources like TriNetX,^29–31^ IBM Explorys,^32^ and Optum.^33^ For studies utilizing UK datasets, the Whitehall II study^34–39^ (n = 8) and UK biobank^40–46^ (n = 7) cohorts were the most frequently used, linked to various UK EHR datasets, including the Hospital Episode Statistics,^47^ Scottish Morbidity Record data,^48^ and Patient Episode Database.^40, 41 46^ Other frequently used databases in the UK studies included the Clinical Practice Research Datalink (n = 6)^49–53^ and the Health Improvement Network (THIN) (n = 4).^21, 54, 55^

### EHR Dataset Sample Size

The studies employed datasets with varying sample sizes, from hundred to millions of patients. Only one study had fewer than 1000 patients.^25^ Twenty-six (32.5%) studies had datasets ranging from 1,000 to 10,000 patients; 46 (57.5%) studies had datasets with 10,000 to one million patients. Seven (8.8%) studies used datasets with over one million patients.

### Outcomes and Measurements

Most studies (n = 67) examined multiple dementia subtypes, including AD, vascular dementia, Lewy body dementia (LBD), frontotemporal dementia (FTD), and mixed dementia. AD was consistently included in all studies, with nine studies exclusively focused on AD. The majority of these studies defined outcomes using standard coding systems, such as ICD codes (81.3%, n = 65), Read codes (11.3%, n = 9), and SNOMED-CT (2.5%, n = 2). Additionally, some studies employed alternative methods, including prescriptions for dementia medications,^16, 18, 26, 33^ cognitive function tests,^25, 42, 56^ referencing the Diagnostic and Statistical Manual of Mental Disorders (Fourth Edition),^22, 57, 58^ screening interviews,^14, 56^ and neuroimaging.^42^

### Risk and Protective Factors

We summarized the analyzed risk factors in the reviewed articles, categorizing medical conditions and interventions into broad disease categories (Table 1). Other risk factors were classified into lifestyle, socioeconomic, environmental, and miscellaneous categories (Table 2).

### Medical conditions

Out of the 80 articles reviewed, 39 (48.8%) explored the interplay between medical conditions and ADRD. Of them, 15 articles focused on cardiovascular and metabolic conditions, 11 on infections, inflammatory and immune-related conditions, 7 on neurological/ophthalmological conditions, 5 on physical function and frailty, 4 on psychiatric conditions, 2 on cancer,^45, 59^ and 4 on other risk factors like kidney disease,^23, 36^ osteoarthritis,^26^ and hip fracture.^17^

*Cardiovascular and metabolic conditions*: A significant finding in our analysis is the association of cardiovascular/metabolic conditions and ADRD risk. Diabetes, examined in several studies,^23, 26, 35, 60, 61^ and its common complication, hypoglycemia, identified as a risk factor,^16, 49, 56, 62^ are noteworthy. Extensive research using EHR data has explored blood pressure’s relationship with ADRD. Hypertension,^23, 61 63, 64^ hypotension^20^ and blood pressure variability,^65^ all contribute to increased ADRD risk. Additional risk factors include coronary artery disease,^23^ stroke,^23^ and hyperlipidemia.^23^ Obesity’s impact is mixed: it has been identified as a risk factor in one study,^53^ suggested to have a potential protective effect in another,^40^ and found to have no impact in a third,^26, 53^ although this may be influenced by factors like age at assessment and frailty in underweight individuals.

#### Infections, inflammatory and immune-related conditions

Hiv^66, 67^ e. coli,^68^ and Covid-19^29^ have been identified as risk factors for ADRD. However, several common infections–such as sepsis, pneumonia, lower respiratory tract infections, urinary tract infections, and skin and soft tissue infections–did not exhibit increased ADRD risk.^42^ Regarding herpes viruses, one study observed a slightly decreased risk of dementia among individuals with symptomatic Herpes Simplex Virus 1 (HSV-1) infections untreated by antivirals and a more pronounced 25% decrease in those treated with antivirals.^69^ Another study detected a minor protective link between Herpes Zoster (HZ) and dementia, particularly in frail individuals and females, and only for mixed or unspecified dementia.^50^ Additionally, the inflammatory/autoimmune disease cluster was associated with elevated ADRD risk,^45^ including inflammatory bowel disease was also found as a risk factor.^32^ Both high urate^40^ and gout^55^ were associated with a decreased risk for ADRD, possibly due to uric acid’s antioxidant effects, which align with observations related to obesity.^22^

#### Psychiatric conditions

The interplay between depression and ADRD remains unclear. While some view depression as a symptom, others see it as a precursor. In our final analysis, three articles explored the link, and all identified a depression as a risk factor for ADRD.^14, 26, 70^ Additionally, psychotic disorders have been reported as a risk factor.^15^

#### Neurological/ophthalmological conditions

The eyes and brain also form crucial nodes in the ADRD risk network. Retinal vascular occlusion is linked to increased ADRD risk.^58^ Visual impairment, assessed by visual acuity, has also been linked to an elevated ADRD risk,^41^ although one study did not find this connection.^71^ The impact of diabetic retinopathy, a complication from diabetes, remains ambiguous, with one study indicating increased risk^72^ and another observed no effect.^73^ Traumatic brain injury^74^ and epilepsy^75^ are identified as risk factors.

#### Physical function and frailty

Frailty metrics are factors to consider in ADRD risk assessment. Underweight is identified as a risk factor for ADRD,^20, 26, 40^ although one study had a different finding.^53^ The protective effect of obesity, sometimes observed, could be related to avoiding the increased risk associated with being underweight.^22^ Low physical function, measured by grip strength and the Short Physical Performance Battery (SPPB), is linked to increased risk.^25^ However, another study did not find an association between physical inactivity or unintentional low caloric intake and ADRD risk.

#### Other medical conditions

Several studies have investigated a miscellany of medical conditions in related to ADRD. Cancer is noteworthy, with one study showing an elevated ADRD risk in the cancer disease cluster.^45^ In contrast, another study found that malignant melanoma and non-melanoma skin cancers were associated with a reduced ADRD risk, suggesting a protective effect.^59^ Additionally, kidney disease,^23, 36^ hip fracture,^17^ and osteoarthritis^26^ were identified as ADRD risk factors.

### Medical interventions

In light of the risk posed by medical conditions to ADRD, researchers have examined various medical interventions to determine if they could mitigate the risk of ADRD. Of the 80 articles assessed, 25 (31.3%) analyzed the association between medical interventions and ADRD. Out of these, 11 were related to cardiovascular and metabolic interventions, 5 to immune, infection, and inflammatory interventions, 4 to psychiatric interventions, 3 to oncology, and 4 to other interventions.

#### Cardiovascular and metabolic-related interventions

Research has focused on treatments targeting cardiovascular and metabolic conditions to reduce ADRD risk. Medications such as rosuvastatin,^76^ telmisartan,^24^ anticoagulants,^52^ and aspirin,^30^ primarily for cardiovascular health, have proven effective in lowering ADRD risk. In diabetes management, metformin showed no association with incident dementia compared to no initial treatment within the first 6 months post-diagnosis.^77^ However, it presented a mild protective effect compared to sulfonylureas.^33, 78^ Conversely, thiazolidinedione monotherapy and combined therapy with metformin reduced ADRD risk compared to metformin alone.^78^ Sodium-glucose co-transporter 2 inhibitors decreased the risk of dementia in patients with atrial fibrillation and type 2 diabetes.^31^ Among surgical interventions, bariatric surgery increased ADRD risk,^79^ while carotid endarterectomy had no discernible impact.^27^

#### Immune, infection and inflammatory-related interventions

Immune, infection and inflammatory-related interventions, such as tumor necrosis factor blocking agent,^80^ methotrexate^21^ and antiherpetic medications,^19^ were found to have protective effects against ADRD, while nonsteroidal anti-inflammatory drugs (NSAIDs)^51^ were observed to increase the risk.

#### Psychiatric-related interventions

Studies had contradictory conclusions on the impact of the selective serotonin reuptake inhibitor (SSRI) antidepressant class on ADRD risk, with one suggesting it as a risk factor^81^ and the other as protective.^76^ Trazodone, another serotonergic antidepressant often used for insomnia, was reported as a neutral factor.^54^

#### Oncology and other Interventions

Androgen deprivation therapy was linked to an increased risk for ADRD in two studies by the same team.^82, 83^ However, aromatase inhibitor therapy and tamoxifen, used for hormone receptor-positive breast cancer, did not show a difference in dementia risk.^84^

### Lifestyle, socioeconomic, psychosocial and environmental factors

EHR data have been utilized to examine the influence of lifestyle, socioeconomic, psychosocial, and environmental factors on ADRD. Out of the reviewed articles, 14 (17.5%) were related to this topic, with 5 articles focused on lifestyles, 5 on socioeconomic factors, 3 on environmental factors, and 2 on psychosocial factors.

#### Lifestyle

Both smoking^26^ and extensive alcohol consumption^28^ were identified as risk factors for ADRD. Conversely, a healthy lifestyle, including no current smoking, moderate alcohol consumption, regular physical activity, healthy diet, adequate sleep duration, less sedentary behavior, and frequent social contact, exhibited a protective effect against ADRD in patients with type II diabetes.^44^ However, diet alone was not found to be protective against ADRD.^37, 85^

#### Socioeconomic factors

Higher education showed neuroprotective effects in two of three studies on education and ADRD risk,^86, 87^ although the third study found no significant correlation.^38^ Neighborhood disadvantage^26, 88^ and low occupational position^38^ were associated to a higher risk of ADRD.

#### Psychosocial factors

Psychosocial factors such as social isolation have been identified as risk factors for ADRD.^46^ In contrast, frequent social contact appears to be a protective factor.^39^ Another metric, the “feeling of loneliness,” was not associated with an increased or decreased risk.^46^

#### Environmental factors

EHR data was used to analyze several environmental risk factors for ADRD. Being born in high stroke mortality states^89^ and exposure to Agent Orange among veterans^90^ were found to be associated with an increased risk of ADRD. Additionally, lithium levels in drinking water were associated with greater risk of dementia in women.^91^

## DISCUSSION

In this scoping review, we conducted comprehensive searches across three major databases to identify studies that utilized EHR data to analyze risk factors associated with cognitive decline. The final selection of 80 articles spans a wide range of risk factors, including medical conditions, interventions, lifestyle, socioeconomic status, psychosocial, and environmental factors. The majority of studied medical conditions were associated with an elevated risk of ADRD, whereas medical interventions addressing these conditions often reduced the ADRD risk. Using large and diverse EHR datasets has enriched the literature on antecedent risk factors for dementia and confirmed findings from smaller sample studies.

Longitudinal EHR data are essential for ADRD research due to the slow progression of the disease. The prolonged latency period between risk factor exposure and clinical symptoms necessitates extended observation to identify early signs and risk factors, facilitating causality assessment.

Utilizing EHR datasets offers numerous benefits for ADRD research. These datasets provide extensive data with a wealth of variables, enabling the exploration of diverse medical conditions and interventions to identify risk and protective factors for cognitive impairment and dementia. These datasets allow simultaneously investigation of multiple potential risk and protective factors while enabling comprehensive adjustments for confounders. Access to large and diverse EHR datasets enhances statistical power,^92^ allowing for the study of rare events and the identification of unique risk profiles and disease trajectories.^59, 90^ These datasets encompass individuals from various backgrounds, facilitating research across different populations and the examination of various disease subtypes and clinical presentation variations. EHR offers rich, detailed clinical data that enable in-depth studies into the clinical aspects and mechanisms of ADRD. Additionally, EHR datasets can confirm and provide unique insights into factors sometimes overlooked or absent in other types of studies.

To facilitate the investigation of risk factors, including socioeconomic aspects, lifestyle, and environmental factors, EHR data are often linked to external datasets using patient identifiers like names, social security numbers, and zip codes. This approach enables the exploration of additional factors and the incorporation of confounding variables from the EHR.

The utilization of EHR data has the potential to help identify new risk factors for ADRD, as well as analyze the traditionally recognized risk factors from a new perspective. Traditional risk factors, such as diabetes,^23, 26^·^35, 60, 61^ hypertension,^23, 61 63, 64^ stroke,^23^ hyperlipidemia,^23^ and traumatic brain injury,^74^ have been confirmed in the studies utilizing EHR data. Additionally, our review presents a list of newly recognized potential risk factors emerging from EHR data analysis. These include but are not limited to, environmental exposures like Agent Orange,^90^ infections such as COVID-19,^29^ and certain surgical procedures previously not associated with ADRD risk. The study by Kim et al,^79^ for example, was the first to find that bariatric surgery increases the risk of ADRD, which contrasts with earlier research that has suggested potential cognitive benefits related to weight loss and metabolic improvement post-surgery. The advent of big data has enabled the identification of these novel risk factors, offering fresh insights into the multifactorial nature of ADRD.

Exploring various populations and EHR datasets reveals inconsistencies in findings on factors like obesity, hypertension, visual impairment, metformin, and underweight, as well as potential conflicts with results from studies not included in this review. The divergent findings underscore the complexity of ADRD risk factors, emphasizing the importance of further research to elucidate these relationships.

Using EHR datasets for ADRD research offers valuable insights but comes with notable limitations. The accuracy and completeness of diagnostic coding in EHRs can vary, impacting the reliability of outcome and exposure classification. Another constraint is the outcome measure heterogeneity and quality in EHR-based studies. Dementia definitions vary, including or excluding subtypes like vascular dementia and LBD, and using different coding systems (ICD, SNOMED CT, READ). Some use cognitive tests with smaller samples, while others rely on ICD codes for larger samples but potentially less specific diagnoses. This diversity in dementia definitions reflects the complexity of diagnosing and classifying cognitive decline and dementia in real-world clinical settings. It affects the identification of cognitive decline risk factors, leading to variability in reported associations. For instance, studies focusing on specific dementia subtypes may reveal unique risk factors that differ from those identified in broader dementia studies. The choice of diagnostic codes and cognitive assessments can also influence the accuracy of dementia identification, thereby affecting the strength and direction of associations between risk factors and cognitive decline. Minimizing variability in outcome measures could substantially enhance the interpretability and comparability of findings in cognitive decline research. Standardizing the criteria for dementia diagnosis across majority of healthcare providers, as opposed to limiting it to a few specialists (dementia experts), could simplify the synthesis of research results and refine the accuracy of associations with risk factor. It could also enhance the detection of subtle or nuanced associations between risk factors and cognitive decline that might be obscured by the current heterogeneity.

EHR-based studies provide valuable insights but do not conclusively establish causality due to the potential influence of uncontrolled confounding variables. Investigations into the link between depression and dementia highlight this challenge. Studies related to diabetes management often fail to distinguish between the cognitive effects of specific diabetes medications and those resulting from overall glycemic control, leaving it unclear if observed benefits stem from particular drugs or general blood sugar management. Additionally, trazodone was found as a risk factor for ADRD; however, the study suggests that the higher incidence of dementia observed among trazodone users might not imply a direct causal relationship but could instead reflect the medication’s use in managing symptoms common in the early stages of cognitive impairment.^54^ Therefore, when interpreting results from those included observational studies, readers should be cautious not to presume a direct causal relationship between the risk factors studied and the outcomes.

Furthermore, the inherent biases in observational studies, including potential confounding, selection bias, and information bias, continue to be pervasive issues. Although most included studies attempted to adjust for known confounders, the possibility of residual confounding cannot be dismissed. Adjusting for confounders in survival models may not be sufficient, especially with numerous confounders or significant covariate overlap between the groups being compared. This can lead to issues such as multicollinearity and overfitting. Advanced statistical methods, including propensity score matching (PSM) and inverse probability weighting (IPW), are often used to reduce bias in the estimation of exposure or treatment effects. Nevertheless, EHR-based studies are not equivalent to randomized controlled clinical trials, the gold standard for establishing causality. Researchers should also consider the context of EHR data collection, including demographic and clinical characteristics of study populations. Variations in healthcare access and utilization across different populations could influence the observed associations. Notably, crucial data, such as information on deaths, may be absent from the EHRs. While some studies have cross-referenced EHR data with external databases to create more comprehensive datasets, not all have followed this approach.

Additional methodological concerns arise in statistical analysis of the included studies. Long follow-up times introduce competing events like death, potentially impacting the event of interest (e.g., AD diagnosis). The widely used Cox model is not suitable for handling competing risks properly, as it treats them as censored, potentially yielding biased results when the assumption of independent censoring is violated. In contrast, the Fine-Gray model estimates covariate effects on the sub-distribution hazard, offering insights into risk and protective factors’ relationships with the event of interest while considering competing risks. Therefore, it is crucial to evaluate study-specific quality indicators, like adherence to the STROBE guidelines, validated outcome measures, and statistical analyses robustness, to prevent overinterpretation of the findings.

Lastly, geographic and demographic constraints exist. Despite the extensive data in EHR systems, research is often localized to specific healthcare systems or geographic locales, limiting generalizability. For instance, unlike UK, the US and other countries appear to underutilize national-level EHR datasets. Despite assess to longitudinal EHR datasets across various healthcare systems and regions, research is often confined to specific EHRs. The VHA dataset, though national, predominantly represent male individuals, poses a demographic limitation. Expanding the use of such comprehensive data sources can provide a more representative sample and enhance research generalizability. The lack of research utilizing large, diverse, national EHR datasets underscore the need for future studies on dementia risk through such resources.

The underdiagnosis of MCI and dementia presents a significant challenge in ADRD research, particularly during early stages. Reliance on EHRs for diagnosis can inadvertently contribute to underreporting, affecting the accuracy of prevalence and incidence rates in the literature. This skewing, due to EHR-based data extraction, might underestimate the true burden of these conditions. Consequently, such underestimation can impact systematic or scoping review findings, altering our understanding of risk factors, disease progression, and intervention effectiveness. Interestingly, individuals frequently interacting with psychiatric services for other conditions are more likely to have cognitive impairment noted in their EHRs compared to those without psychiatric conditions. Therefore, studies that consider psychiatric conditions as risk factors for ADRD particularly require careful interpretation.

### Future directions

The analysis of the articles suggests several avenues for future investigation using EHR data.

1. Medical interventions: The impact of medical treatments on reducing cognitive decline in the context of various medical conditions remains unclear. There is a lack of research on pharmacological and surgical effects compared to studies on medical conditions and ADRD. Future research should prioritize studying the relationship between medical interventions and cognitive decline more broadly.
2. Explore overlooked factors: investigate additional risk or protective factors, like genetic markers (e.g., apolipoprotein E, presenilin 1 and 2, and amyloid precursor protein), environmental toxins (e.g., lead, pesticides),^93^ mild traumatic brain injury,^94^ endocrine factors (such as hypothyroidism), sleep disturbance (like sleep apnea or chronic sleep deprivation),^7, 95^ bilingualism,^96^ vitamin and nutritional deficiencies,^97^ and the microbiome (e.g., gut microbiome).^98^
3. Clinical notes and AI: Almost all the reviewed articles have used data from structured fields of the EHR. Certain conditions and symptoms (e.g., hearing loss, sleep disturbances) that are not consistently captured in structured EHR data may require the examination of clinical notes to identify them, often necessitating AI and natural language processing.
4. Early cognitive decline: While the existing literature primary focuses on dementia or AD, fewer studies address the early onset of AD and the initial stages of cognitive decline, such as mild cognitive impairment and subjective cognitive decline.
5. Diversify study populations: Most EHR-based studies have focused on populations with well-defined medical conditions like diabetes, hypertension, cancer, and HIV. To advance research, it’s essential to include a broader range of specific groups, such as sexual and gender minorities,^99, 100^ indigenous populations, those resilient to cognitive decline, and various psychiatric cohorts.
6. Database integration: Integrating diverse EHR database across institutions and locations, like the UK’s national datasets, can expand study populations and enhance research generalizability, which is currently underutilized in the US and other countries.
7. Data linkage: EHRs lack some data and require linkage with other datasets,^101^ including insurance claims, genetics, socioeconomic status,^102^ lifestyle, crime, and environmental factors (e.g., air pollution, wildfires, climate change, toxic chemicals).

### Limitations

This review has several limitations to acknowledge. First, our search was constrained to three databases, potentially missing relevant studies in other sources. Second, our search term, focused on titles and abstracts, might have overlooked articles using different terminology or mentioning EHR components (e.g., clinical notes) in the methods section. Third, we didn’t perform a bias assessment for included observational studies, which is important considering biases in EHR data collection and outcome measures. Fourth, this review doesn’t aim to provide a comprehensive overview of ADRD risk factors; instead, it focuses on what has been studied using EHR data. Finally, we refrained from conducting a meta-analysis due to variations in adjusted confounders among studies, complicating cross-study comparisons.

## CONCLUSION

EHR data, with its rich and diverse longitudinal real-world information, provides substantial insights into the medical conditions, interventions, lifestyle, socioeconomic, and environmental factors associated with ADRD risk. Looking ahead, research should focus on diversifying study populations and integrating EHR data across geographical locations and with non-EHR datasets. There is also a need to enhance the extraction of information from unstructured text to explore a broader range of risk factors for ADRD.

## Figures and Tables

**Table 1:** Summary of medical conditions and interventions from EHR-based studies in related to the risk of Alzheimer’s disease and related dementias.

	Medical conditions (n = 39)		Medical interventions (n = 25)	
Categories	No. of articles	Risk Factors/Exposures	No. of articles	Risk Factors/Exposures
Cardiovascular and metabolic	15	Diabetes↑^23, 26, 35, 60, 61^ Hypoglycemia in diabetic patients↑^16, 49, 56, 62^ Hypertension↑^23, 61, 63,64^↔^26^ Hypotension ↑^20^ High blood pressure variability ↑^65^ Coronary artery disease↑^23^ Stroke ↑^23^ Hyperlipidemia ↑^23^ Dyslipidemia ↔^26^ Obesity ↑^53^ ↓^40^ ↔^26^	11	Antihypertensive medications ↔^26^ Aspirin ↓^30^ Statins ↔^26^ Rosuvastatin ↓^76^ Anticoagulant ↓^52^a Telmisartan ↓^24^ Metformin ↔^77^↓^33^b Thiazolidinedione↓^78^c Sulfonylurea ↑^33,78^c Metformin and thiazolidinedione dual therapy ↓^78^c Sodium-glucose co-transporter 2 inhibitors ↓^31^ Carotid endarterectomy↔^27^ Bariatric surgery↑^79^
Infections, inflammatory, and immune-related	11	HIV ↑^66, 67^ E. coli ↑^68^e Herpes zoster ↓^50^ Covid-^19^ ↑^29^ Symptomatic herpes simplex virus infection ↓^69^ Common infections(sepsis, pneumonia, other LRTIs, UTIs and SSTIs)↔^42^ Inflammatory/autoimmune conditions ↑^45^ Inflammatory Bowel Disease ↑^32^ High urate ↓^40^ Gout ↓^55^	5	Tumor necrosis factor blocking agent ↓^32, 80^ Methotrexate ↓^21, 80^ NSAIDs ↑^51^ Antiherpetic medication ↓^19^ Immunomodulators ↓^32^
Neurological/ophthalmological conditions	7	Retinal vascular occlusion↑^58^ ↔^71^ Visual impairment ↑^41^ Diabetic retinopathy ↑^72^↔^73^ Traumatic brain injury ↑^74^ Epilepsy ↑^75^	1	Cataract extraction ↓^57^
Physical function and frailty	5	Low physical function ↑^25^ Physical inactivity ↔^53^ Underweight ↑^20, 26, 40^ ↔^53^ Low caloric intake ↔^53^		n/a
Psychiatric	4	Depression ↑^14, 26, 70^ Psychotic disorder ↑^15^	4	SSRI ↑^81^ ↓^76^ Trazodone ↑^54^e Benzodiazepines↔^22^
Oncology	2	Cancer ↑^45^ Skin cancer ↓^59^	3	Androgen deprivation therapy↑^82, 83^ Aromatase inhibitor therapy versus tamoxifen ↔^84^d
Other	4	Kidney disease ↑^23, 36^ Hip fracture ↑^17^ Osteoarthritis ↑^26^	3	β-antagonists ↓^18^ Vitamin D ↓^43^ Omeprazole ↓^76^

**Table 2:** Summary of lifestyles, socioeconomic, psychosocial, environmental and other factors from EHR-based studies in related to the risk of Alzheimer’s disease and related dementias.

Categories	No. of articles	Risk Factors/Exposures
**Lifestyle, socioeconomic, psychosocial, environmental risk factors (n = 14)**		
Lifestyle	5	Diet ↔^37, 85^ Healthy lifestyle ↓^44^ Smoking ↑^26^ Alcohol consumption ↑^28^
Socioeconomic	5	High education ↓^86, 87^ ↔^38^ Neighborhood disadvantage ↑^26, 88^ Low occupational position ↑^38^
Psychosocial	2	Social isolation ↑^46^ Social contact ↓^39^ Feeling of loneliness ↔^46^
Environmental	3	Birth in high stroke mortality states ↑^89^ Agent orange ↑^90^ Lithium level in drinking water ↑^91^
**Others (n = 8)**		
Others	8	Plasma protein ↑^34^ Lower testosterone ↑^13^ Higher brain age ↑^103^ Low Childhood IQ ↑^104^ ICU admission ↑^105^ Hispanic race ↑^61^ Sex ↔^26^ CRP genotype ↔^45^ Apolipoprotein E (APOE) genotype ↔^45^

## Data Availability

This article is a literature review and does not involve the generation of new data. All data and materials used in this review are derived from publicly available sources and previously published literature. References to these sources are provided within the manuscript.

## Abbreviations

HER: electronic health records
AD: Alzheimer’s disease
MCI: mild cognitive impairment
ADRD: Alzheimer’s disease and related dementias
STROBE: Strengthening the Reporting of Observational Studies in Epidemiology
VHA: Veterans Health Administration
LBD: Lewy body dementia
FTD: frontotemporal dementia
HSV-1: symptomatic Herpes Simplex Virus 1
HZ: Herpes Zoster
SPPB: Short Physical Performance Battery
NSAIDs: nonsteroidal anti-inflammatory drugs
SSRI: selective serotonin reuptake inhibitor
PSM: propensity score matching
IPW: inverse probability weighting

## References

[1] Jack CR Jr, Bennett DA, Blennow K, NIA-AA research framework: toward a biological definition of Alzheimer’s disease. Alzheimer’s & Dementia 2018; 14: 535–562. 10.1016/j.jalz.2018.02.018 PMC5958625 29653606

[2] Sperling RA, Aisen PS, Beckett LA, Toward defining the preclinical stages of Alzheimer’s disease: Recommendations from the National Institute on Aging-Alzheimer’s Association workgroups on diagnostic guidelines for Alzheimer’s disease. Alzheimer’s & dementia 2011; 7: 280–292. 10.1016/j.jalz.2011.03.003 PMC3220946 21514248

[3] Association As. 2023 Alzheimer’s disease facts and figures. Alzheimer’s & dementia 2023; 19. DOI: 10.1002/alz.13016. 36918389

[4] Veitch DP, Weiner MW, Aisen PS, Using the Alzheimer’s Disease Neuroimaging Initiative to improve early detection, diagnosis, and treatment of Alzheimer’s disease. Alzheimers Dement 2022; 18: 824–857. 2021/09/29. DOI: 10.1002/alz.12422. 34581485 PMC9158456

[5] Dagley A, LaPoint M, Huijbers W, Harvard aging brain study: dataset and accessibility. Neuroimage 2017; 144: 255–258. 25843019 10.1016/j.neuroimage.2015.03.069 PMC4592689

[6] Wolters FJ, Segufa RA, Darweesh SKL, Coronary heart disease, heart failure, and the risk of dementia: A systematic review and meta-analysis. Alzheimers Dement 2018; 14: 1493–1504. 2018/03/02. DOI: 10.1016/j.jalz.2018.01.007. 29494808

[7] Shi L, Chen SJ, Ma MY, Sleep disturbances increase the risk of dementia: A systematic review and meta-analysis. Sleep Med Rev 2018; 40: 4–16. 2017/09/12. DOI: 10.1016/j.smrv.2017.06.010. 28890168

[8] Kuiper JS, Zuidersma M, Oude Voshaar RC, Social relationships and risk of dementia: A systematic review and meta-analysis of longitudinal cohort studies. Ageing Res Rev 2015; 22: 39–57. 2015/05/10. DOI: 10.1016/j.arr.2015.04.006. 25956016

[9] Page MJ, McKenzie JE, Bossuyt PM, The PRISMA 2020 statement: an updated guideline for reporting systematic reviews. International journal of surgery 2021; 88: 105906. 33789826 10.1016/j.ijsu.2021.105906

[10] Knottnerus A and Tugwell P. STROBE--a checklist to Strengthen the Reporting of Observational Studies in Epidemiology. J Clin Epidemiol 2008; 61: 323. 2008/03/04. DOI: 10.1016/j.jclinepi.2007.11.006. 18313555

[11] Cox DR. Regression models and life-tables. Journal of the Royal Statistical Society: Series B (Methodological) 1972; 34: 187–202.

[12] Fine JP and Gray RJ. A proportional hazards model for the subdistribution of a competing risk. Journal of the American statistical association 1999; 94: 496–509.

[13] Ford AH, Yeap BB, Flicker L, Sex hormones and incident dementia in older men: The health in men study. Psychoneuroendocrinology 2018; 98: 139–147. 2018/08/26. DOI: 10.1016/j.psyneuen.2018.08.013. 30144781

[14] Almeida OP, Hankey GJ, Yeap BB, Depression as a risk factor for cognitive impairment in later life: the Health In Men cohort study. Int J Geriatr Psychiatry 2016; 31: 412–420. 2015/08/19. DOI: 10.1002/gps.4347. 26280254

[15] Almeida OP, Ford AH, Hankey GJ, Risk of dementia associated with psychotic disorders in later life: the health in men study (HIMS). Psychol Med 2019; 49: 232–242. 2018/03/23. DOI: 10.1017/s003329171800065x. 29564993

[16] Lee CH, Lui DTW, Cheung CYY, Different glycaemia-related risk factors for incident Alzheimer’s disease in men and women with type 2 diabetes-A sex-specific analysis of the Hong Kong diabetes database. Diabetes Metab Res Rev 2021; 37: e3401. 2020/09/02. DOI: 10.1002/dmrr.3401. 32870568

[17] Hsu WWQ, Zhang X, Sing CW, Hip Fracture as a Predictive Marker for the Risk of Dementia: A Population-Based Cohort Study. J Am Med Dir Assoc 2022; 23: 1720 e1721–1720 e1729. 2022/08/22. DOI: 10.1016/j.jamda.2022.07.013. 35988591

[18] Cui S, Fang F, Cui P, Associations between the use of beta-adrenoceptor acting drugs and the risk of dementia in older population. Front Neurol 2022; 13: 999666. 2023/01/10. DOI: 10.3389/fneur.2022.999666. 36619918 PMC9813664

[19] Schnier C, Janbek J, Williams L, Antiherpetic medication and incident dementia: Observational cohort studies in four countries. Eur J Neurol 2021; 28: 1840–1848. 2021/03/04. DOI: 10.1111/ene.14795. 33657269

[20] Perera G, Rijnbeek PR, Alexander M, Vascular and metabolic risk factor differences prior to dementia diagnosis: a multidatabase case-control study using European electronic health records. BMJ Open 2020; 10: e038753. 2020/11/17. DOI: 10.1136/bmjopen-2020-038753. PMC7668358 33191253

[21] Newby D, Prieto-Alhambra D, Duarte-Salles T, Methotrexate and relative risk of dementia amongst patients with rheumatoid arthritis: a multi-national multi-database case-control study. Alzheimers Res Ther 2020; 12: 38. 2020/04/08. DOI: 10.1186/s13195-020-00606-5. 32252806 PMC7137292

[22] Hafdi M, Hoevenaar-Blom MP, Beishuizen CRL, Association of Benzodiazepine and Anticholinergic Drug Usage With Incident Dementia: A Prospective Cohort Study of Community-Dwelling Older Adults. J Am Med Dir Assoc 2020; 21: 188–193.e183. 2019/07/14. DOI: 10.1016/j.jamda.2019.05.010. 31300339

[23] Kuo SC, Lai SW, Hung HC, Association between comorbidities and dementia in diabetes mellitus patients: population-based retrospective cohort study. J Diabetes Complications 2015; 29: 1071–1076. 2015/08/04. DOI: 10.1016/j.jdiacomp.2015.06.010. 26233574

[24] Liu CH, Sung PS, Li YR, Telmisartan use and risk of dementia in type 2 diabetes patients with hypertension: A population-based cohort study. PLoS Med 2021; 18: e1003707. 2021/07/20. DOI: 10.1371/journal.pmed.1003707. 34280191 PMC8289120

[25] Papadopoulos E, Abu Helal A, Berger A, Objective measures of physical function and their association with cognitive impairment in older adults with cancer prior to treatment. J Geriatr Oncol 2022; 13: 1141–1148. 2022/07/26. DOI: 10.1016/j.jgo.2022.07.007. 35879200

[26] Pham ANQ, Lindeman C, Voaklander D, Risk factors for incidence of dementia in primary care practice: a retrospective cohort study in older adults. Fam Pract 2022; 39: 406–412. 2021/12/16. DOI: 10.1093/fampra/cmab168. 34910126 PMC9155170

[27] Halliday A, Sneade M, Björck M, Editor’s Choice - Effect of Carotid Endarterectomy on 20 Year Incidence of Recorded Dementia: A Randomised Trial. Eur J Vasc Endovasc Surg 2022; 63: 535–545. 2022/03/12. DOI: 10.1016/j.ejvs.2021.12.041. 35272949

[28] Kivimaki M, Singh-Manoux A, Batty GD, Association of Alcohol-Induced Loss of Consciousness and Overall Alcohol Consumption With Risk for Dementia. JAMA Netw Open 2020; 3: e2016084. 2020/09/10. DOI: 10.1001/jamanetworkopen.2020.16084. 32902651 PMC7489835

[29] Wang L, Davis PB, Volkow ND, Association of COVID-19 with New-Onset Alzheimer’s Disease. J Alzheimers Dis 2022; 89: 411–414. 2022/08/02. DOI: 10.3233/jad-220717. 35912749 PMC10361652

[30] Gorenflo MP, Davis PB, Kendall EK, Association of Aspirin Use with Reduced Risk of Developing Alzheimer’s Disease in Elderly Ischemic Stroke Patients: A Retrospective Cohort Study. J Alzheimers Dis 2023; 91: 697–704. 2022/12/12. DOI: 10.3233/jad-220901. 36502331 PMC11388024

[31] Proietti R, Rivera-Caravaca JM, López-Gálvez R, Cerebrovascular, Cognitive and Cardiac Benefits of SGLT2 Inhibitors Therapy in Patients with Atrial Fibrillation and Type 2 Diabetes Mellitus: Results from a Global Federated Health Network Analysis. J Clin Med 2023; 12 2023/04/28. DOI: 10.3390/jcm12082814. PMC10142574 37109151

[32] Aggarwal M, Alkhayyat M, Abou Saleh M, Alzheimer Disease Occurs More Frequently In Patients With Inflammatory Bowel Disease: Insight From a Nationwide Study. J Clin Gastroenterol 2023; 57: 501–507. 2022/04/27. DOI: 10.1097/mcg.0000000000001714. 35470286

[33] Newby D, Linden AB, Fernandes M, Comparative effect of metformin versus sulfonylureas with dementia and Parkinson’s disease risk in US patients over 50 with type 2 diabetes mellitus. BMJ Open Diabetes Res Care 2022; 10 2022/09/16. DOI: 10.1136/bmjdrc-2022-003036. PMC9478804 36109050

[34] Lindbohm JV, Mars N, Walker KA, Plasma proteins, cognitive decline, and 20-year risk of dementia in the Whitehall II and Atherosclerosis Risk in Communities studies. Alzheimers Dement 2022; 18: 612–624. 2021/08/03. DOI: 10.1002/alz.12419. 34338426 PMC9292245

[35] Barbiellini Amidei C, Fayosse A, Dumurgier J, Association Between Age at Diabetes Onset and Subsequent Risk of Dementia. Jama 2021; 325: 1640–1649. 2021/04/28. DOI: 10.1001/jama.2021.4001. 33904867 PMC8080220

[36] Singh-Manoux A, Oumarou-Ibrahim A, Machado-Fragua MD, Association between kidney function and incidence of dementia: 10-year follow-up of the Whitehall II cohort study. Age Ageing 2022; 51 2022/01/22. DOI: 10.1093/ageing/afab259. PMC8782607 35061870

[37] Akbaraly TN, Singh-Manoux A, Dugravot A, Association of Midlife Diet With Subsequent Risk for Dementia. Jama 2019; 321: 957–968. 2019/03/13. DOI: 10.1001/jama.2019.1432. 30860560 PMC6436698

[38] Rusmaully J, Dugravot A, Moatti JP, Contribution of cognitive performance and cognitive decline to associations between socioeconomic factors and dementia: A cohort study. PLoS Med 2017; 14: e1002334. 2017/06/27. DOI: 10.1371/journal.pmed.1002334. 28650972 PMC5484463

[39] Sommerlad A, Sabia S, Singh-Manoux A, Association of social contact with dementia and cognition: 28-year follow-up of the Whitehall II cohort study. PLoS Med 2019; 16: e1002862. 2019/08/03. DOI: 10.1371/journal.pmed.1002862. 31374073 PMC6677303

[40] Cao Z, Xu C, Yang H, Associations of BMI and Serum Urate with Developing Dementia: A Prospective Cohort Study. J Clin Endocrinol Metab 2020; 105 2020/09/13. DOI: 10.1210/clinem/dgaa638. 32918088

[41] Littlejohns TJ, Hayat S, Luben R, Visual Impairment and Risk of Dementia in 2 Population-Based Prospective Cohorts: UK Biobank and EPIC-Norfolk. J Gerontol A Biol Sci Med Sci 2022; 77: 697–704. 2021/11/01. DOI: 10.1093/gerona/glab325. 34718565 PMC8974347

[42] Muzambi R, Bhaskaran K, Rentsch CT, Are infections associated with cognitive decline and neuroimaging outcomes? A historical cohort study using data from the UK Biobank study linked to electronic health records. Transl Psychiatry 2022; 12: 385. 2022/09/16. DOI: 10.1038/s41398-022-02145-z. 36109502 PMC9478085

[43] Geng T, Lu Q, Wan Z, Association of serum 25-hydroxyvitamin D concentrations with risk of dementia among individuals with type 2 diabetes: A cohort study in the UK Biobank. PLoS Med 2022; 19: e1003906. 2022/01/14. DOI: 10.1371/journal.pmed.1003906. 35025861 PMC8797194

[44] Wang B, Wang N, Sun Y, Association of Combined Healthy Lifestyle Factors With Incident Dementia in Patients With Type 2 Diabetes. Neurology 2022; 99: e2336–e2345. 2022/09/15. DOI: 10.1212/wnl.0000000000201231. 36104282

[45] Khondoker M, Macgregor A, Bachmann MO, Multimorbidity pattern and risk of dementia in later life: an 11-year follow-up study using a large community cohort and linked electronic health records. J Epidemiol Community Health 2023; 77: 285–292. 2023/03/09. DOI: 10.1136/jech-2022-220034. 36889910

[46] Elovainio M, Lahti J, Pirinen M, Association of social isolation, loneliness and genetic risk with incidence of dementia: UK Biobank Cohort Study. BMJ Open 2022; 12: e053936. 2022/02/25. DOI: 10.1136/bmjopen-2021-053936. PMC8867309 35197341

[47] Herbert A, Wijlaars L, Zylbersztejn A, Data resource profile: hospital episode statistics admitted patient care (HES APC). International journal of epidemiology 2017; 46: 1093–1093i. 28338941 10.1093/ije/dyx015 PMC5837677

[48] Harley K and Jones C. Quality of Scottish morbidity record (SMR) data. Health bulletin 1996; 54: 410–417. 8936810

[49] Mehta HB, Mehta V and Goodwin JS. Association of Hypoglycemia With Subsequent Dementia in Older Patients With Type 2 Diabetes Mellitus. J Gerontol A Biol Sci Med Sci 2017; 72: 1110–1116. 2016/10/28. DOI: 10.1093/gerona/glw217. 27784724 PMC5861972

[50] Warren-Gash C, Williamson E, Shiekh SI, No evidence that herpes zoster is associated with increased risk of dementia diagnosis. Ann Clin Transl Neurol 2022; 9: 363–374. 2022/02/17. DOI: 10.1002/acn3.51525. 35170873 PMC8935278

[51] Dregan A, Chowienczyk P and Armstrong D. Patterns of anti-inflammatory drug use and risk of dementia: a matched case-control study. Eur J Neurol 2015; 22: 1421–1428. 2015/07/16. DOI: 10.1111/ene.12774. 26177125

[52] Cadogan SL, Powell E, Wing K, Anticoagulant prescribing for atrial fibrillation and risk of incident dementia. Heart 2021; 107: 1898–1904. 2021/10/15. DOI: 10.1136/heartjnl-2021-319672. 34645643 PMC8600601

[53] Floud S, Simpson RF, Balkwill A, Body mass index, diet, physical inactivity, and the incidence of dementia in 1 million UK women. Neurology 2020; 94: e123–e132. 2019/12/20. DOI: 10.1212/WNL.0000000000008779. 31852815 PMC6988985

[54] Brauer R, Lau WCY, Hayes JF, Trazodone use and risk of dementia: A population-based cohort study. PLoS Med 2019; 16: e1002728. 2019/02/06. DOI: 10.1371/journal.pmed.1002728. 30721226 PMC6363148

[55] Lu N, Dubreuil M, Zhang Y, Gout and the risk of Alzheimer’s disease: a population-based, BMI-matched cohort study. Ann Rheum Dis 2016; 75: 547–551. 2015/03/06. DOI: 10.1136/annrheumdis-2014-206917. 25739830 PMC4560667

[56] Hendrie HC, Zheng M, Li W, Glucose level decline precedes dementia in elderly African Americans with diabetes. Alzheimers Dement 2017; 13: 111–118. 2016/10/30. DOI: 10.1016/j.jalz.2016.08.017. 27793691 PMC5318260

[57] Lee CS, Gibbons LE, Lee AY, Association Between Cataract Extraction and Development of Dementia. JAMA Intern Med 2022; 182: 134–141. 2021/12/07. DOI: 10.1001/jamainternmed.2021.6990. 34870676 PMC8649913

[58] Lee CS, Lee ML, Gibbons LE, Associations Between Retinal Artery/Vein Occlusions and Risk of Vascular Dementia. J Alzheimers Dis 2021; 81: 245–253. 2021/03/23. DOI: 10.3233/jad-201492. 33749651 PMC8168611

[59] Ibler E, Tran G, Orrell KA, Inverse association for diagnosis of Alzheimer’s disease subsequent to both melanoma and non-melanoma skin cancers in a large, urban, single-centre, Midwestern US patient population. J Eur Acad Dermatol Venereol 2018; 32: 1893–1896. 2018/03/25. DOI: 10.1111/jdv.14952. 29573497 PMC6153078

[60] Doney ASF, Bonney W, Jefferson E, Investigating the Relationship Between Type 2 Diabetes and Dementia Using Electronic Medical Records in the GoDARTS Bioresource. Diabetes Care 2019; 42: 1973–1980. 2019/08/09. DOI: 10.2337/dc19-0380. 31391202

[61] Nagar SD, Pemu P, Qian J, Investigation of hypertension and type 2 diabetes as risk factors for dementia in the All of Us cohort. Sci Rep 2022; 12: 19797. 2022/11/18. DOI: 10.1038/s41598-022-23353-z. 36396674 PMC9672061

[62] Hendrie HC, Zheng M, Lane KA, Changes of glucose levels precede dementia in African-Americans with diabetes but not in Caucasians. Alzheimers Dement 2018; 14: 1572–1579. 2018/04/22. DOI: 10.1016/j.jalz.2018.03.008. 29678640 PMC6192866

[63] Gilsanz P, Mayeda ER, Glymour MM, Female sex, early-onset hypertension, and risk of dementia. Neurology 2017; 89: 1886–1893. 2017/10/06. DOI: 10.1212/wnl.0000000000004602. 28978656 PMC5664296

[64] Abell JG, Kivimaki M, Dugravot A, Association between systolic blood pressure and dementia in the Whitehall II cohort study: role of age, duration, and threshold used to define hypertension. Eur Heart J 2018; 39: 3119–3125. 2018/06/15. DOI: 10.1093/eurheartj/ehy288. 29901708 PMC6122131

[65] Whiteley WN, Gupta AK, Godec T, Long-Term Incidence of Stroke and Dementia in ASCOT. Stroke 2021; 52: 3088–3096. 2021/07/02. DOI: 10.1161/strokeaha.120.033489. 34192893 PMC8478091

[66] Bobrow K, Xia F, Hoang T, HIV and risk of dementia in older veterans. Aids 2020; 34: 1673–1679. 2020/07/24. DOI: 10.1097/qad.0000000000002597. 32701576

[67] Lam JO, Hou CE, Hojilla JC, Comparison of dementia risk after age 50 between individuals with and without HIV infection. AIDS 2021; 35: 821–828. 2021/01/05. DOI: 10.1097/QAD.0000000000002806. 33394681 PMC7969394

[68] Katz J and Gao H. The Alzheimer-E. coli Axis: What Can We Learn from an Electronic Health Record Platform. J Alzheimers Dis 2021; 84: 717–721. 2021/09/28. DOI: 10.3233/jad-215004. 34569963

[69] Young-Xu Y, Powell EI, Zwain GM, Symptomatic Herpes Simplex Virus Infection and Risk of Dementia in US Veterans: a Cohort Study. Neurotherapeutics 2021; 18: 2458–2467. 2021/07/11. DOI: 10.1007/s13311-021-01084-9. 34244925 PMC8804043

[70] Gilsanz P, Schnaider Beeri M, Karter AJ, Depression in type 1 diabetes and risk of dementia. Aging Ment Health 2019; 23: 880–886. 2018/04/11. DOI: 10.1080/13607863.2018.1455167. 29634288 PMC6179940

[71] Chan AX, Bakhoum CY, Bangen KJ, Relationship between Retinal Vascular Occlusions and Cognitive Dementia in a Large Cross-Sectional Cohort. Am J Ophthalmol 2021; 226: 201–205. 2021/02/03. DOI: 10.1016/j.ajo.2021.01.026. 33529587 PMC9227960

[72] Exalto LG, Biessels GJ, Karter AJ, Severe diabetic retinal disease and dementia risk in type 2 diabetes. J Alzheimers Dis 2014; 42 Suppl 3: S109–117. 2014/03/15. DOI: 10.3233/jad-132570. 24625797 PMC4373321

[73] Rodill LG, Exalto LG, Gilsanz P, Diabetic Retinopathy and Dementia in Type 1 Diabetes. Alzheimer Dis Assoc Disord 2018; 32: 125–130. 2017/12/21. DOI: 10.1097/wad.0000000000000230. 29261519 PMC5963957

[74] Barnes DE, Kaup A, Kirby KA, Traumatic brain injury and risk of dementia in older veterans. Neurology 2014; 83: 312–319. 2014/06/27. DOI: 10.1212/wnl.0000000000000616. 24966406 PMC4115602

[75] Kim Y, Lhatoo S, Zhang GQ, Temporal phenotyping for transitional disease progress: An application to epilepsy and Alzheimer’s disease. J Biomed Inform 2020; 107: 103462. 2020/06/21. DOI: 10.1016/j.jbi.2020.103462. 32562896 PMC7374015

[76] Xu J, Wang F, Zang C, Comparing the effects of four common drug classes on the progression of mild cognitive impairment to dementia using electronic health records. Sci Rep 2023; 13: 8102. 2023/05/20. DOI: 10.1038/s41598-023-35258-6. 37208478 PMC10199021

[77] Salas J, Morley JE, Scherrer JF, Risk of incident dementia following metformin initiation compared with noninitiation or delay of antidiabetic medication therapy. Pharmacoepidemiol Drug Saf 2020; 29: 623–634. 2020/05/05. DOI: 10.1002/pds.5014. 32363681 PMC8457517

[78] Tang X, Brinton RD, Chen Z, Use of oral diabetes medications and the risk of incident dementia in US veterans aged ≥60 years with type 2 diabetes. BMJ Open Diabetes Res Care 2022; 10 2022/10/12. DOI: 10.1136/bmjdrc-2022-002894. PMC9472121 36220195

[79] Kim J, Kelley J, Kleinschmit K, Development of dementia in patients who underwent bariatric surgery. Surg Endosc 2023; 37: 3507–3521. 2022/12/30. DOI: 10.1007/s00464-022-09837-z. 36581785

[80] Zhou M, Xu R, Kaelber DC, Tumor Necrosis Factor (TNF) blocking agents are associated with lower risk for Alzheimer’s disease in patients with rheumatoid arthritis and psoriasis. PLoS One 2020; 15: e0229819. 2020/03/24. DOI: 10.1371/journal.pone.0229819. 32203525 PMC7089534

[81] Wang C, Gao S, Hendrie HC, Antidepressant Use in the Elderly Is Associated With an Increased Risk of Dementia. Alzheimer Dis Assoc Disord 2016; 30: 99–104. 2015/08/22. DOI: 10.1097/wad.0000000000000103. 26295747 PMC4760914

[82] Nead KT, Gaskin G, Chester C, Androgen Deprivation Therapy and Future Alzheimer’s Disease Risk. J Clin Oncol 2016; 34: 566–571. 2015/12/09. DOI: 10.1200/jco.2015.63.6266. 26644522 PMC5070576

[83] Nead KT, Gaskin G, Chester C, Association Between Androgen Deprivation Therapy and Risk of Dementia. JAMA Oncol 2017; 3: 49–55. 2016/10/14. DOI: 10.1001/jamaoncol.2016.3662. 27737437

[84] Bromley SE, Matthews A, Smeeth L, Risk of dementia among postmenopausal breast cancer survivors treated with aromatase inhibitors versus tamoxifen: a cohort study using primary care data from the UK. J Cancer Surviv 2019; 13: 632–640. 2019/07/20. DOI: 10.1007/s11764-019-00782-w. 31321612 PMC6776493

[85] Flores AC, Jensen GL, Mitchell DC, Prospective Study of Diet Quality and the Risk of Dementia in the Oldest Old. Nutrients 2023; 15 2023/03/12. DOI: 10.3390/nu15051282. PMC10005581 36904280

[86] Soh Y, Whitmer RA, Mayeda ER, State-Level Indicators of Childhood Educational Quality and Incident Dementia in Older Black and White Adults. JAMA Neurol 2023; 80: 352–359. 2023/02/14. DOI: 10.1001/jamaneurol.2022.5337. 36780143 PMC9926357

[87] Hayes-Larson E, Ikesu R, Fong J, Association of Education With Dementia Incidence Stratified by Ethnicity and Nativity in a Cohort of Older Asian American Individuals. JAMA Netw Open 2023; 6: e231661. 2023/03/07. DOI: 10.1001/jamanetworkopen.2023.1661. 36877520 PMC9989900

[88] Becerril A, Pfoh ER, Hashmi AZ, Racial, ethnic and neighborhood socioeconomic differences in incidence of dementia: A regional retrospective cohort study. J Am Geriatr Soc 2023 2023/03/18. DOI: 10.1111/jgs.18322. 36928611

[89] Gilsanz P, Mayeda ER, Glymour MM, Association Between Birth in a High Stroke Mortality State, Race, and Risk of Dementia. JAMA Neurol 2017; 74: 1056–1062. 2017/08/02. DOI: 10.1001/jamaneurol.2017.1553. 28759663 PMC5691590

[90] Martinez S, Yaffe K, Li Y, Agent Orange Exposure and Dementia Diagnosis in US Veterans of the Vietnam Era. JAMA Neurol 2021; 78: 473–477. 2021/01/26. DOI: 10.1001/jamaneurol.2020.5011. 33492338 PMC7835948

[91] Duthie AC, Hannah J, Batty GD, Low-level lithium in drinking water and subsequent risk of dementia: Cohort study. Int J Geriatr Psychiatry 2023; 38: e5890. 2023/02/08. DOI: 10.1002/gps.5890. 36747488

[92] Casey JA, Schwartz BS, Stewart WF, Using Electronic Health Records for Population Health Research: A Review of Methods and Applications. Annu Rev Public Health 2016; 37: 61–81. 2015/12/17. DOI: 10.1146/annurev-publhealth-032315-021353. 26667605 PMC6724703

[93] Yan D, Zhang Y, Liu L, Pesticide exposure and risk of Alzheimer’s disease: a systematic review and meta-analysis. Sci Rep 2016; 6: 32222. 2016/09/02. DOI: 10.1038/srep32222. 27581992 PMC5007474

[94] Clark E, Faruque S, Mutebi C, Investigating the relationship between mild traumatic brain injury and Alzheimer’s disease and related dementias: a systematic review. J Neurol 2022; 269: 4635–4645. 2022/06/02. DOI: 10.1007/s00415-022-11186-9. 35648232

[95] Guay-Gagnon M, Vat S, Forget MF, Sleep apnea and the risk of dementia: A systematic review and meta-analysis. J Sleep Res 2022; 31: e13589. 2022/04/03. DOI: 10.1111/jsr.13589. 35366021

[96] Brini S, Sohrabi HR, Hebert JJ, Bilingualism Is Associated with a Delayed Onset of Dementia but Not with a Lower Risk of Developing it: a Systematic Review with Meta-Analyses. Neuropsychol Rev 2020; 30: 1–24. 2020/02/10. DOI: 10.1007/s11065-020-09426-8. 32036490 PMC7089902

[97] Zhou J, Sun Y, Ji M, Association of Vitamin B Status with Risk of Dementia in Cohort Studies: A Systematic Review and Meta-Analysis. J Am Med Dir Assoc 2022; 23: 1826 e1821–1826 e1835. 2022/07/03. DOI: 10.1016/j.jamda.2022.05.022. 35779574

[98] Coradduzza D, Sedda S, Cruciani S, Age-Related Cognitive Decline, Focus on Microbiome: A Systematic Review and Meta-Analysis. International Journal of Molecular Sciences 2023; 24: 13680. 37761988 10.3390/ijms241813680 PMC10531012

[99] Hua Y, Wang L, Nguyen V, A deep learning approach for transgender and gender diverse patient identification in electronic health records. J Biomed Inform 2023; 147: 104507. 2023/10/02. DOI: 10.1016/j.jbi.2023.104507. 37778672 PMC10687838

[100] Romanelli RJ, Rosenblatt AS, Marcum ZA, Cognitive Impairment in Sexual and Gender Minority Groups: A Scoping Review of the Literature. LGBT Health 2023 2023/10/12. DOI: 10.1089/lgbt.2023.0095. 37824757

[101] DeBord DG, Carreon T, Lentz TJ, Use of the “Exposome” in the Practice of Epidemiology: A Primer on -Omic Technologies. Am J Epidemiol 2016; 184: 302–314. 2016/08/16. DOI: 10.1093/aje/kwv325. 27519539 PMC5025320

[102] Kind AJH and Buckingham WR. Making Neighborhood-Disadvantage Metrics Accessible - The Neighborhood Atlas. N Engl J Med 2018; 378: 2456–2458. 2018/06/28. DOI: 10.1056/NEJMp1802313. 29949490 PMC6051533

[103] Biondo F, Jewell A, Pritchard M, Brain-age is associated with progression to dementia in memory clinic patients. Neuroimage Clin 2022; 36: 103175. 2022/09/11. DOI: 10.1016/j.nicl.2022.103175. 36087560 PMC9467894

[104] Russ TC, Hannah J, Batty GD, Childhood Cognitive Ability and Incident Dementia: The 1932 Scottish Mental Survey Cohort into their 10th Decade. Epidemiology 2017; 28: 361–364. 2017/02/06. DOI: 10.1097/ede.0000000000000626. 28151744 PMC5381709

[105] Schulte PJ, Warner DO, Martin DP, Association Between Critical Care Admissions and Cognitive Trajectories in Older Adults. Crit Care Med 2019; 47: 1116–1124. 2019/05/21. DOI: 10.1097/ccm.0000000000003829. 31107280 PMC6629515

